# Complexity Analysis of Escher’s Art

**DOI:** 10.3390/e21060553

**Published:** 2019-05-31

**Authors:** António M. Lopes, J. A. Tenreiro Machado

**Affiliations:** 1 UISPA–LAETA/INEGI, Faculty of Engineering, University of Porto, Rua Dr. Roberto Frias, 4200-465 Porto, Portugal; 2 Institute of Engineering, Polytechnic of Porto, Department of Electrical Engineering, R. Dr. António Bernardino de Almeida, 431, 4249-015 Porto, Portugal

**Keywords:** complexity, information theory, multidimensional scaling, art

## Abstract

Art is the output of a complex system based on the human spirit and driven by several inputs that embed social, cultural, economic and technological aspects of a given epoch. A solid quantitative analysis of art poses considerable difficulties and reaching assertive conclusions is a formidable challenge. In this paper, we adopt complexity indices, dimensionality-reduction and visualization techniques for studying the evolution of Escher’s art. Grayscale versions of 457 artworks are analyzed by means of complexity indices and represented using the multidimensional scaling technique. The results are correlated with the distinct periods of Escher’s artistic production. The time evolution of the complexity and the emergent patterns demonstrate the effectiveness of the approach for a quantitative characterization of art.

## 1. Introduction

During human history, artists conceived harmonies of objects and forms in their works [1]. Artworks are manifestations of the artists’ creativity, reflecting their thoughts and culture [2]. Often we have a glimpse of a kind of mathematical exercise embedded in the artworks [3,4,5,6,7,8,9]. It is well known the symbiosis of art and science produced by Leonardo da Vinci [10], or the magic complexity of the music composed by Bach [11]. The fascination of many artists/scientists for art/mathematics is ubiquitous in human history [12,13,14]. Nowadays, we can take advantage of computational algorithms to stimulate synergies between art and science.

Art is a token of a complex system produced by mankind and influenced by a plethora of social, cultural, economic and technological inputs that interact in time and space. The study of artworks may help interpreting the world and the human mind. However, an assertive analysis of art poses conceptual and practical difficulties and reaching quantitative conclusions represents a huge challenge.

The tools to analyze complex systems have been successfully adopted in different areas, including economics, life and social sciences [15], with the objective of finding fundamental principles and universalities that govern the systems’ dynamics [16,17]. Complexity is helpful to quantitatively describe nonlinear systems and to detect changes in their dynamics.

Several complexity indices have been adopted to analyze art, namely entropy [18,19], Kolmogorov complexity [20,21], fractal dimension [22,23], and others [24,25]. Such indices are not independent, but they capture different aspects of the system state, complementing each other and leading to a deeper assessment of the subject under study [26]. The quantitative analysis of art dates back to 1933, when Birkhoff proposed an aesthetic measure as the ratio between order (i.e., number of regularities) and complexity (i.e., number of elements) of an image. Nevertheless, only recently quantitative techniques were applied, impelled by the availability of digital data and the development of computational tools. Taylor et al. [22] verified that Jackson Pollock’s (1912–1956) dripped patterns are fractals and that the fractal dimension of the paintings increased over the course of his artistic career. Dodgson [18] adopted the concepts of entropy and correlation to describe Bridget Riley’s (1961–2012) stripe paintings. Cucker [3] suggested that geometry is an important source of rules for artistic creation. Wallraven et al. [27] used multidimensional scaling (MDS) and clustering techniques to categorize paintings. Kim et al. [28] analyzed a large database of images, finding that the color-usage distribution is remarkably different among historical periods of western paintings. Lee et al. [29] examined almost 180 thousand paintings, focusing on the evolution of the color contrast. Among other findings, they observed a sudden increase in the diversity of color contrast after 1850. Machado and Lopes [20] studied paintings from the viewpoint of information theory and fractional calculus. Sigaki et al. [19] addressed the local order patterns of almost 140 thousand artwork images using complexity indices and observed a clear and robust time evolution.

Escher (1898–1972) is one of the most celebrated modern graphic artists [30,31]. He is known for his works in woodcuts, lithographs and mezzotints representing fantastic, unusual and impossible objects, with various perspectives, generating optical illusions. Escher is considered a mathematical artist, especially geometric, influenced by his relationships with mathematicians and by its own interests and abilities in mathematics. His artworks explore aspects such as infinity, perspective, symmetry, reflection, hyperbolic geometry, truncated and stellated polyhedra, and tessellations [32,33]. Escher made about 450 lithographs, woodcuts and wood engravings, and over 2000 drawings and sketches. Despite some open discussion about his 54-year long artistic career, Escher’s work is classified in several periods tightly related to the places where he lived in. We observe five distinct periods [34]:

- $P_{1}$—The early work period (1916–1922)—Escher lived in Arnhem and Haarlem, in the Netherlands. Most pieces of this period consist of woodcuts and linocuts produced when he was a student. The artwork is varied in theme, going from portraits to drawings that hint at cubism. Escher developed the linocut printmaking technique and learned to represent figures in black and white. One aspect of this process was to learn to ‘think backwards’, since images carved into woodblocks for printing must be carved backwards, as though seen in a mirror (e.g., ‘Skull’, c. 1920).
- $P_{2}$—The Italian period (1922–1937)—Escher lived in Italy from 1924 to 1935. During those years he traveled across the country and created a large portfolio of lithographs and wood engravings based on drawings of Italian buildings, landscapes and seascapes (e.g., ‘Castrovalva’, c. 1930). In this period Escher was focused in representing the reality and his portfolio includes also plants, animals and portraits. An early attempt to make some different drawings, with the interpenetration of distinct worlds, came just in the final phase of this period (e.g., ‘Candle Mirror’, c. 1934).
- $P_{3}$—The metamorphosis period (1937–1945)—Escher left Italy in 1935, lived in Switzerland and Belgium between 1935 and 1941, and went back to the Netherlands in 1941. In 1936 Escher revisited Spain and traveled to Alhambra and Cordoba. This trip inspired him to the subject of tessellations with great impact in his art. During this period he focused on representing the world as how it could be instead as how it really was. The artworks include cycles and the transformation of 3-dim into 2-dim forms. The symmetry and the perfect fit of shapes are also characteristic marks of this period (e.g., ‘Day and Night’, c. 1938).
- $P_{4}$—The subordinated to perspective period (1946–1956)—in this period Escher worked with engravings, using unusual and multiple viewpoints, vanishing points and perspectives. Some works suggest the infinity of space through multiple vanishing points and bundles of straight lines. Escher stressed the sense of depth through the use of colors, progressively blurred throughout the pictures and creating the idea of an aerial perspective (e.g., ‘Depth’, c. 1955). He also demonstrated interest in geometric solids due to his studies in mineralogy and crystallography.
- $P_{5}$—The approximation to infinity period (1956–1970)—in this period Escher made several engravings that have as central theme the infinity, where he explored ideas from hyperbolic geometry (e.g., ‘Circle Limit III’, c. 1959). This period is also characterized by the production of impossible figures, with perspectives, reflections, conflicts of dimension, illusion, and the shape of space (e.g., ‘Art Gallery 1956’, c. 1956, ‘Waterfall’, c. 1961).

In this paper we adopt complexity indices, dimensionality reduction and visualization techniques for studying the evolution of Escher’s art. In the first phase, 457 artworks, produced between 1916 and 1969, were converted into digital format, discretized and represented in grayscale. The images were then processed by computing six distinct complexity indices, whose evolution was correlated with the periods of the artist’s career. In a second phase, a MDS algorithm is adopted for visualizing complexity. The MDS was fed with dissimilarity information calculated with two different distance measures. The MDS maps were interpreted under the light of the emerging clusters and correlated with the periods of Escher’s art. It should be noted that other indices can be used for quantifying complexity and different techniques can be adopted for dimensionality reduction, clustering and visualization. We can mention, for example, the use of time–frequency signal processing and hierarchical clustering for studying tidal data [35], the Lempel–Ziv complexity, sample entropy, signal harmonics power ratio, and fractal dimension for analyzing temperature time series [26], and information theory, fractional calculus and hierarchical clustering for studying art [20].

Considering these ideas, Section 2 introduces the mathematical background, emphasizing the concept of complexity and the MDS technique. Section 3 analyzes the evolution of Escher’s art in the perspective of six complexity indices. Section 4 uses MDS for dimensionality reduction and data visualization, and interprets the generated maps in the perspective of the periods of the artist’s career. Finally, Section 5 presents the conclusions.

## 2. Mathematical Background

### 2.1. Classic Information Indices

The information theory proposed by Shannon [36] was recently adopted in the study of complex systems [37,38].

Let us consider a discrete 1-dim random variable *X* with sample space $\left\{x_{1},\ldots ,x_{i},\ldots ,x_{N}\right\}$ and probability distribution P(X). An event, $x_{i}$, with probability of occurrence $P(x_{i})$ has the information content:

(1) $$h\left[P\left(x_{i}\right)\right]=-\log P\left(x_{i}\right).$$

The Shannon entropy is the arithmetic average, or expected value, of $h\left[P\left(x_{i}\right)\right]$:

(2) $$H\left(X\right)=E\left[-\log P\left(x_{i}\right)\right]=\sum_{i=1}^{N}-P\left(x_{i}\right)\log P\left(x_{i}\right),$$

where the operator $E\left(\cdot \right)$ represents the expected value.

The joint entropy of a two-dimensional discrete random variable (X,Y) with sample spaces $\left\{x_{1},\ldots ,x_{i},\ldots ,x_{N}\right\}$ and $\left\{y_{1},\ldots ,y_{j},\ldots ,y_{M}\right\}$, and joint probability distribution P(X,Y) is [39]:

(3) $$S\left(X,Y\right)=\sum_{i=1}^{N}\sum_{j=1}^{M}-P\left(x_{i},y_{j}\right)\log P\left(x_{i},y_{j}\right).$$

The mutual information of (X,Y), with marginal probability distributions P(X) and P(Y), respectively, is given by [40,41]:

(4) $$I\left(X;Y\right)=\sum_{i=1}^{N}\sum_{j=1}^{M}P\left(x_{i},y_{j}\right)\log\frac{P\left(x_{i},y_{j}\right)}{P\left(x_{i}\right)P\left(y_{j}\right)}$$

and assesses the information shared by (X,Y). When *X* and *Y* are independent, there is no shared information between them, and the mutual information is I(X;Y)=0.

In the follow-up, the complexity indices S(X,Y) and I(X;Y) will be interpreted as state variables and the locus (S,I) will be called the entropy-mutual information plane.

The Jensen–Shannon divergence measures the similarity between two probability distributions P(X) and P(Y) and is defined as [41]:

(5) $$JSD\left[P(X)\|P(Y)\right]=\frac{1}{2}\left[\sum_{i=1}^{N}P\left(x_{i}\right)\log P\left(x_{i}\right)+\sum_{i=1}^{N}P\left(y_{i}\right)\log P\left(y_{i}\right)\right]-\sum_{i=1}^{N}P\left(z_{i}\right)\log P\left(z_{i}\right),$$

where *X* and *Y* are random variables with sample spaces $\left\{x_{1},\ldots ,x_{i},\ldots ,x_{N}\right\}$ and $\left\{y_{1},\ldots ,y_{i},\ldots ,y_{N}\right\}$, and $Z=\frac{1}{2}\left(X+Y\right)$.

### 2.2. Permutation Entropy and Statistical Complexity

The permutation entropy (PE) was originally proposed as a robust index to assess the complexity of time series [42].

For a time series, $\left\{x_{k}:k=1,\ldots ,W\right\}$, $x_{k}\in R$, we define two parameters: the embedding dimension, d≥2, d∈N, and the embedding delay, τ∈N, that represent the length of the time series partitioning sequences, and the separation time between their elements, respectively. Let us denote by $\Psi =\{\Pi_{1},\ldots ,\Pi_{d!}\}$ the set of all possible permutations of the ordinals {1,…,d}, and by [I] the Iverson bracket [43], such that $I=\left\{\begin{array}{cc} 1, & \mathrm{if}\;I\;\mathrm{is}\;\mathrm{true}\\ 0, & \mathrm{if}\;I\;\mathrm{is}\;\mathrm{false} \end{array}\right.$. The procedure for calculating PE is as follows:

1. For each k=1,…,K, with K=W−(d−1)τ,
  1.1. Compose the sequence $\left\{x_{k},x_{k+\tau },\ldots ,x_{k+(d-1)\tau }\right\}$;
  1.2. Construct the 2×d dimensional array $\left[\begin{array}{cccc} x_{k} & x_{k+\tau } & \ldots & x_{k+(d-1)\tau }\\ 1 & 2 & \ldots & d \end{array}\right]$;
  1.3. Sort the array by increasing order of the elements in the first row;
  1.4. Denote by $\pi_{k}$ the sequence of numbers in the second row of the sorted array;
2. Compute the probability distribution $P=[p_{1},p_{2},\ldots ,p_{d!}]$, where $p_{i}=\frac{1}{K}\sum_{k=1}^{K}\left[\pi_{k}=\Pi_{i}\right]$, i=1,…,d!;
3. Calculate $PE\left(P\right)=\frac{1}{\log d!}\sum_{i=1}^{d!}-p_{i}\log p_{i}$.

The value of PE lies in the interval 0≤PE≤1. The lower value PE=0 indicates that the time series is regular or predictable, while the upper value PE=1 corresponds to a random time series. The embedding dimension must be chosen such that W≫d! in order to obtain reliable values of PE. For practical purposes the values d∈{3,…,7} and τ=1 are suggested [42].

For two-dimensional data, as is the case of images, the generalization of the procedure is straightforward. Let us consider a greyscale image represented by the $N_{x}\times N_{y}$ matrix *A*. We define four parameters: two for the embedding dimensions, $d_{x},d_{y}\geq 2$, with $d_{x},d_{y}\in N$, and two for the embedding delays, $\tau_{x},\tau_{y}\in N$. The symbolic sequences for calculating the probabilities, $P=[p_{1},p_{2},\ldots ,p_{(d_{x}d_{y})!}]$, are now obtained from the spatial information in overlapping $d_{x}\times d_{y}$ dimensional submatrices. We have a total of $(d_{x}d_{y})!$ permutations of the ordinals $\left\{1,\ldots ,d_{x}d_{y}\right\}$ and we choose $d_{x}$ and $d_{y}$ such that $N_{x}N_{y}\ll \left(d_{x}d_{y}\right)!$ for having reliable values for PE (for details see [44,45]). For images, the PE is a measure of ‘randomness’ in the layout of the pixels. If the pixels appear in a random (in the same) order, then PE→1 (PE→0).

Another complexity measure is the statistical complexity (*C*) proposed in [46,47]:

(6) $$C\left(P\right)=\frac{1}{\kappa }\cdot JSD\left[P\|U\right]\cdot PE\left(P\right),$$

where $JSD\left[P\|U\right]$ is the Jensen–Shannon divergence between $P=[p_{1},p_{2},\ldots ,p_{(d_{x}d_{y})!}]$ and the uniform distribution $U=\left[u_{1},u_{2},\ldots ,u_{(d_{x}d_{y})!}\right]=\frac{1}{(d_{x}d_{y})!}\cdot \left[1,1,\ldots ,1\right]$, whereas

(7) $$\kappa =\max_{P}\left\{JSD\left[P\|U\right]\right\}=-\frac{1}{2}\left[\frac{(d_{x}d_{y})!+1}{(d_{x}d_{y})!}\log\left[(d_{x}d_{y})!+1\right]+\log\left[(d_{x}d_{y})!\right]-2\log\left[2(d_{x}d_{y})!\right]\right].$$

is a normalization constant.

Often the locus (PE,C) is adopted for characterizing data, being called as the complexity-entropy plane [46,48].

### 2.3. Kolmogorov Complexity-Based Indices

The Kolmogorov complexity, K(A), of an object *A* provides a measure of information independently of any probabilistic assumptions about the data sequences in *A*. The complexity K(A) is defined as the size of the shortest program that, given an empty object at its input, computes *A* in an universal computer and then stops [49,50,51]. The exact value of K(A) is not computable [49,50,51,52,53,54]. Therefore, approximations based on the Lempel–Ziv [55], linguistic [56] and compression [57] algorithms are used to obtain the upper bounds K(A).

Lossless compression algorithms approximate K(A) by the size of the compressed object, K(A)≈size{Φ(A)}, where Φ(·) denotes compression [50,51]. However, for obtaining a good approximation, the compressor has to be ‘normal’. This means that, given the object *A* and the concatenation of *A* with itself, AA, the compressor has to generate compressed objects such that size[Φ(A)]≈size[Φ(AA)] [50,51]. For having a complexity index independent of size[A] we use the complexity ratio (CR):

(8) $$CR=\frac{\mathrm{size}[\Phi (A)]}{\mathrm{size}[A]}.$$

The information distance between two objects $\left\{A_{1},A_{2}\right\}$ can be computed using the conditional Kolmogorov complexity, $K(A_{1}|A_{2})$. This corresponds to the size of the shortest program to compute $A_{1}$, given that $A_{2}$ is provided [58,59]. Therefore, when we have almost similar objects $A_{1}$ and $A_{2}$, the task is less complex and the size of the program is smaller. The inequality $K\left(A_{1}|A_{2}\right)\leq K\left(A_{1}\right)$ always holds and the normalized information distance, NID, is formulated as an universal metric [58]:

(9) $$NID\left(A_{1},A_{2}\right)=\frac{\max\{K\left(A_{1}|A_{2}\right),K\left(A_{2}|A_{1}\right)\}}{\max\{K\left(A_{1}\right),K\left(A_{2}\right)\}}.$$

The NID is a distance and thus it satisfies the conditions:

1. $NID(A_{1},A_{2})\geq 0$; moreover, we have (i) $NID(A_{1},A_{2})=0$, if and only if $A_{1}=A_{2}$; and (ii) $NID(A_{1},A_{2})=1$, if and only if $A_{2}$ is an empty object (non-negativity);
2. $NID\left(A_{1},A_{2}\right)=NID\left(A_{2},A_{1}\right)$ (symmetry);
3. $NID\left(A_{1},A_{2}\right)\leq NID\left(A_{1},A_{3}\right)+NID\left(A_{3},A_{2}\right)$ (triangle inequality).

The NID is based on K(·) and it is not computable [58]. To surpass this limitation, the normalized compression distance:

(10) $$NCD\left(A_{1},A_{2}\right)=\frac{\mathrm{size}\left[\Phi \left(A_{1}A_{2}\right)\right]-\min\left\{\mathrm{size}\left[\Phi \left(A_{1}\right)\right],\mathrm{size}\left[\Phi \left(A_{2}\right)\right]\right\}}{\max\{\mathrm{size}\left[\Phi \left(A_{1}\right)\right],\mathrm{size}\left[\Phi \left(A_{2}\right)\right]\}}$$

was proposed for approximating the NID [60,61]. The NCD is nonnegative, and we have $0<NCD(A_{1},A_{2})<1+\epsilon$, where ϵ>0 denotes the error introduced by the compressor [61,62].

Stemming from (10), we propose the complexity index $NCD_{R}\left(A,R\right)$, where *R* represents a reference object of the same size of *A*. We tested for *R* pixels with random noise and all-identical shade of gray. The results showed that $NCD_{R}\left(A,R\right)$ is quite insensitive to the type of *R*, since it consists of a normalized distance. The locus $\left(CR,NCD_{R}\right)$ will be designated by compression distance-ratio plane.

### 2.4. Multidimensional Scaling

The MDS is a technique for dimensionality reduction, clustering and computational visualization of multidimensional data [63,64,65,66,67,68,69]. Given *L* objects $x_{i}$, i=1,…,L, in a *r*-dim space and a measure of dissimilarity between the *i*th and *j*th objects, $\delta_{ij}\left(x_{i},x_{j}\right)$, the procedure starts by calculating a L×L symmetric matrix, $\Delta =[\delta_{ij}]$ of object-to-object dissimilarities. The matrix Δ gives the input information to the MDS numerical algorithm. The MDS represents objects by means of points located in a *q*-dim space (q<r) at distances $\theta_{ij}$, and iterates multiple configurations in order to maximize a fitness function and to achieve a map of points that approximates the original ones. By other words, the MDS calculates the matrix of distances $\Theta =[\theta_{ij}]$ that try to mimic $\Delta =[\delta_{ij}]$. A fitness function widely used is the raw stress:

(11) $$R={\left[\theta_{ij}-f\left(\delta_{ij}\right)\right]}^{2},$$

where f(·) denotes some linear or non-linear transformation.

The MDS interpretation follows the patterns of points obtained in the MDS locus. Two similar (dissimilar) objects are shown as two points that are close to (far from) each other. Thus, the MDS interpretation does not follow neither the coordinates of the points, nor the shape of the clusters. In fact, we can translate, rotate and magnify the map, since the object-to-object distances are identical. Moreover, the MDS axes have neither units nor special physical meaning.

The MDS quality can be quantified by means of the Shepard and stress plots. The Shepard diagram compares $\theta_{ij}$ and $\delta_{ij}$, for a particular value of *q*. A narrow scattering of the points represents a good fit between $\theta_{ij}$ and $\delta_{ij}$. The stress diagram represents the locus of R versus *q*. Usually users adopt q=2 or q=3, because such values allow a direct visualization and establish a compromise between achieving low values of R and *q*.

## 3. Complexity of Escher’s Art

This Section addresses the evolution of Escher’s art through time in the perspective of six complexity indices. Section 3.1 characterizes the data set and the conversion scheme of the artwork into digital format. Section 3.2 analyzes the time evolution of the complexity indices. Section 3.3 discusses the complexity of Escher’s art under the light of the loci (S,I), (PE,C) and $\left(CR,NCD_{R}\right)$.

### 3.1. Data Description

**Figure 1:**
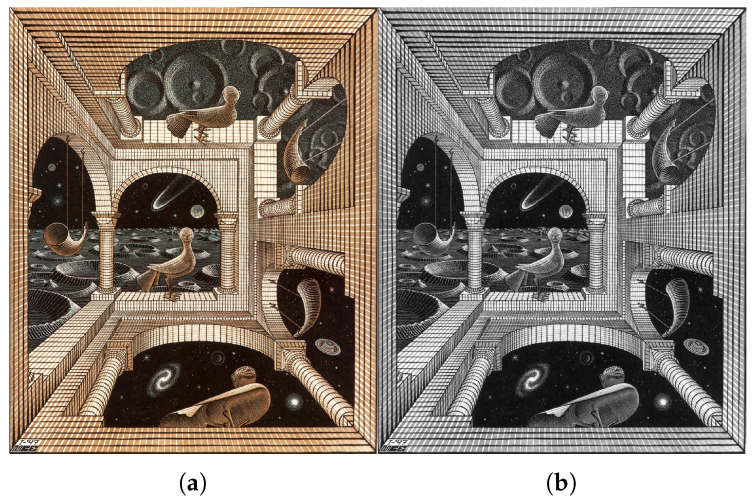
The artwork ‘Another World II’ (c. 1947): (**a**) color image; (**b**) grayscale image.

The study involved 457 artworks created by Escher between the years 1916 and 1969. The digital images were obtained from the *visual arts encyclopedia*, at the website www.wikiart.org, on 5 March 2019. WikiArt is one of the largest visual arts databases available online for free. Each image file, stored in JPEG format, is read into a $N_{x}\times N_{y}\times N_{z}$ dimensional matrix, *A*. For $N_{z}=3$, *A* represents color images and its elements are 8-bit integers in the range between 0 and 255, corresponding to red, green and blue (RGB) intensities. The color images are converted to grayscale and represented by two-dimensional matrices ($N_{z}=1$), where the elements of *A* denote values between black and white (Gr), generated by the ITU-R BT.601 RGB to gray conversion scheme, Gr=0.2989R+0.5870G+0.1140B. This pre-processing yields some loss of information in the originals, not only in terms of color and texture, but also about the three-dimensional textural surface of the painting. However, since most Escher paintings are black and white or with shades of gray, the procedure, necessary for reducing the volume of information and convert all works into an uniform format, does not compromise significantly the analysis. For example, Figure 1 depicts the ‘Another World II’ (c. 1947), and its corresponding grayscale image.

### 3.2. Time Evolution of the Complexity Indices

Here the artworks are characterized by means of the complexity indices in the set $\left\{S,I,PE,C,CR,NCD_{R}\right\}$. Their time evolution is then correlated with the Escher’s artistic periods $P_{i}$, i=1,…,5.

**Figure 2:**
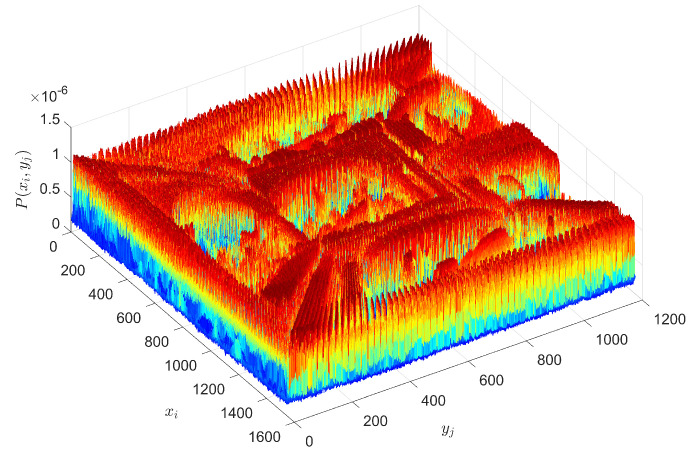
The two-dimensional histogram of the grayscale version of the ‘Another World II’ (c. 1947) by Escher.

For calculating *S* and *I* the probabilities $P(x_{i},y_{j})$ are obtained from the matrices $A=[a_{ij}]$, $i=1,2,\ldots ,N_{x}$, $j=1,2,\ldots ,N_{y}$, through the proportion $P\left(x_{i},y_{j}\right)=\frac{a_{ij}}{\sum_{i=1}^{N_{x}}\sum_{j=1}^{N_{y}}a_{ij}}$, where $x_{i}$ and $y_{i}$ are the *i*-th row and *j*-th column. Therefore $P(x_{i},y_{i})$ represents a normalized version of an image *A* in the perspective of probability. Figure 2 presents an illustrative example, showing $P(x_{i},y_{j})$ for the grayscale version of the artwork ‘Another World II’.

For determining PE and *C*, we use the parameters $d_{x}=d_{y}=2$ and $\tau_{x}=\tau_{y}=1$, which were adjusted by numeric simulations. For computing CR and $NCD_{R}$ we adopt the Windows implementation of the gzip compressor, version 1.3.12 (built upon the Lempel-Ziv coding algorithm LZ77), while the reference for the $NCD_{R}$ is a white image.

**Figure 3:**
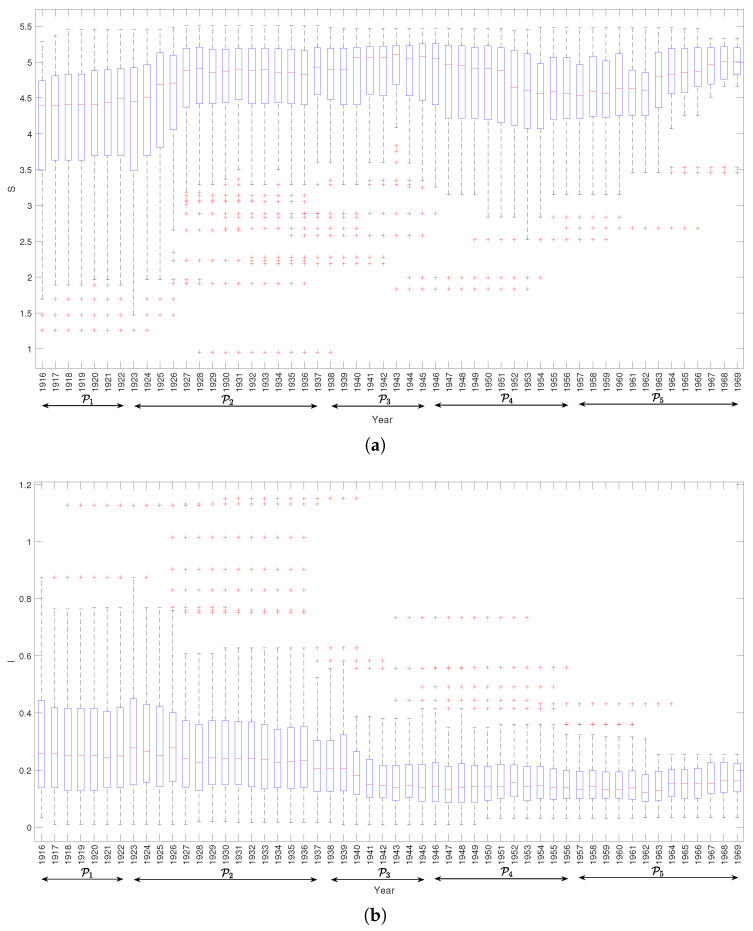
Two complexity indices versus time for the period 1916–1969: (**a**) *S*; (**b**) *I*.

Figure 3 depicts the *S* and *I* versus time, using box plots, for the period 1916–1969. In each box, the central mark indicates the median, the bottom and top edges correspond to the 25 and 75 percentiles, respectively, the whiskers span between the extreme data points (not considering the outliers), and the outliers are depicted using the marker ‘+’. The complexity indices were calculated considering artworks within five-year windows centered at the time stamp. Numerical experiments showed that this value was a good compromise for obtaining a readable and detailed graphical representation. Lower values increase the detail, but blur the charts, while higher values tend to filter too much the data and details are lost. We note that *S* has higher variations than *I*, discriminating better between periods. Indeed, we can find a relationship between the time evolution of *S* and *I* and the different periods of the Escher’s artistic career, even knowing that this division is neither rigidly defined, nor absolutely consensual [34].

We can mention five emerging periods:

- $P_{1}$ (1916–1922) the joint entropy *S* and the mutual information *I* stay approximately constant;
- $P_{2}$ (1922–1937) the *S* takes a leap up and then stabilizes, while *I* reveals a slight increase, with some oscillation, and then stabilizes;
- $P_{3}$ (1937–1945) the value of *S* increases and *I* decreases;
- $P_{4}$ (1946–1956) the *S* decreases considerable and reaches a new local minimum, while the value of *I* remains approximately constant;
- $P_{5}$ (1956–1970) the value of *S* reveals an increasing trend, while *I* changes only a little bit, revealing a slight increase just at the end of the period.

For the other complexity indices we reach similar conclusions and, therefore, the corresponding box plots are omitted.

In synthesis, the complexity indices unveil direct correspondence with Escher’s artistic periods. However, we note in the box plots some dispersion and outliers that may rise interesting questions. For example, what is it about the outliers that make the complexity indices to exhibit fluctuation? Should the outliers be interpreted as anomalies? What is it about the quality of the original images? Since they are from a public database, is the quality reliable? Does it have influence in the results?

### 3.3. Loci of the Complexity Indices

**Figure 4:**
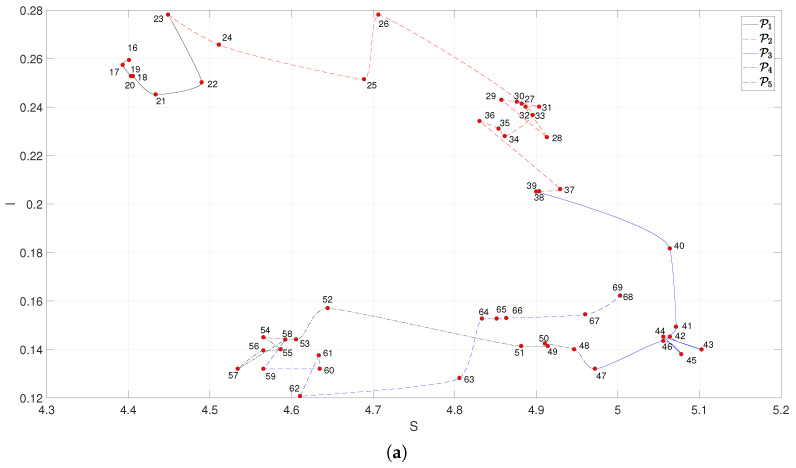

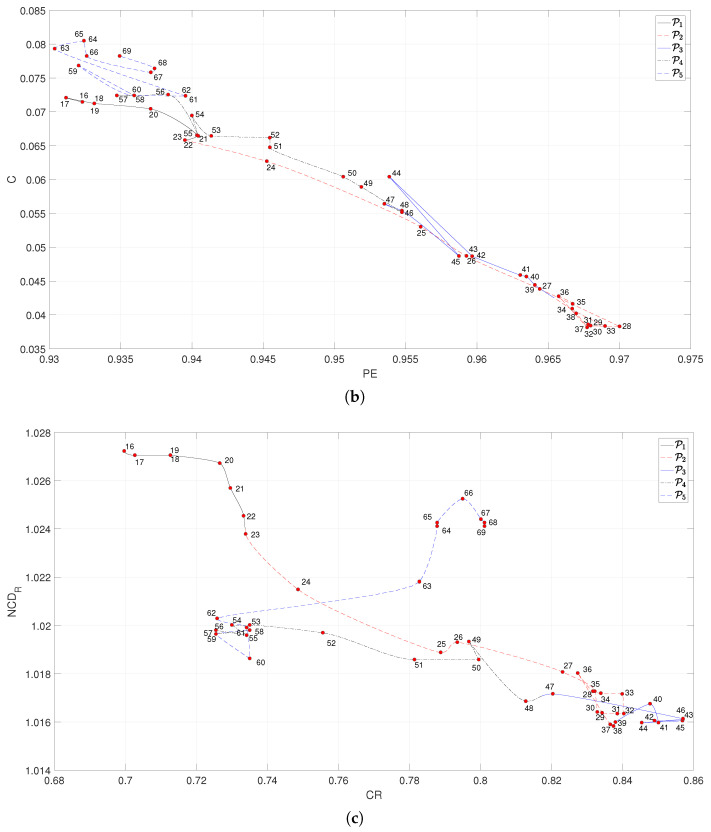
The three complexity loci for the period 1916–1969: (**a**) (S,I); (**b**) (PE,C); (**c**) $\left(CR,NCD_{R}\right)$.

We analyzed the loci (S,I), (PE,C) and $\left(CR,NCD_{R}\right)$ to assess the complexity of Escher’s art. The resulting curves are depicted in Figure 4, where the labels denote the two last digits of the years (form 1916 to 1969) and the points correspond to the medians of the complexity indices of artworks within year-centered five-year length windows. This windowing procedure hides some details, but gives a clear picture of the global locus.

Analyzing the locus (S,I) (Figure 4a), we verify that during $P_{1}$ the complexity has a very limited evolution, for $P_{2}$ it has a large excursion, for $P_{3}$ it changes direction, for $P_{4}$ it has another large excursion and, finally for $P_{5}$ it evolves almost in opposite direction with respect to the former period. It is also interesting to see that between two consecutive periods $P_{i}$ and $P_{i+1}$, (i=2,3,4) we always has a tangle revealing the artist search for the new direction of work. For the locus (PE,C) (Figure 4b), we verified that the complexity indices were strongly correlated and, therefore, the five periods of Escher’s career appear somewhat overlapped. Therefore, the exercise of discriminating $P_{i}$, i=1,…,5, had some limitations. Moreover, we did not find an obvious presence of the inter-period tangles observed in Figure 4a. For $\left(CR,NCD_{R}\right)$ (Figure 4c), we observed again a good match between the artistic periods of Escher and the locus. Furthermore, we noted also the presence of the inter-period tangles.

## 4. MDS Visualization of Complexity

In this Section we use the MDS technique for visualizing complexity. In Section 4.1 the input for the MDS is the dissimilarity information between artworks computed with the NCD (10). In Section 4.2 the MDS is fed with the dissimilarity information between the six complexity indices $\left\{S,I,PE,C,CR,NCD_{R}\right\}$ calculated with the Euclidean distance.

### 4.1. MDS Visualization of Complexity Based on the NCD

**Figure 5:**
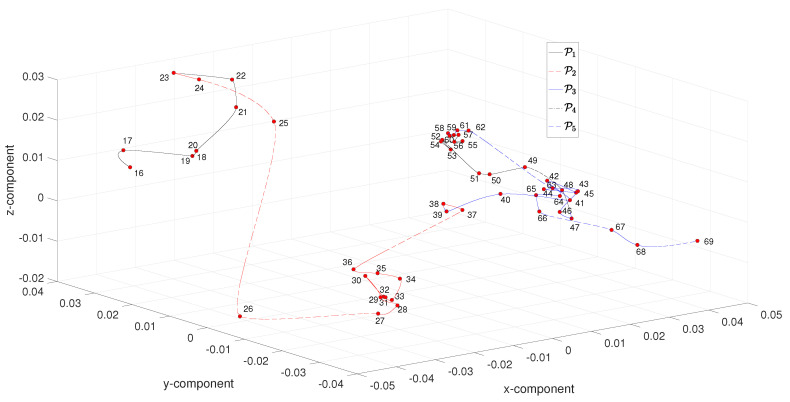
The multidimensional scaling (MDS) three-dimensional map obtained with $\Delta_{NCD}$ for the period 1916–1969.

We compute the 457×457 dimensional matrix $\Delta_{NCD}=\left[NCD\left(A_{i},A_{j}\right)\right]$, where $NCD(A_{i},A_{j})$ is given by (10) and denotes the dissimilarity between the artworks $A_{i}$ and $A_{j}$, i,j=1,…,457. The matrix $\Delta_{NCD}$ is used as the input to the MDS numerical scheme. Since the MDS outputs a large number of points, we post-process the results by calculating the medians of the (x,y,z) coordinates that correspond to artworks within year-centered five-year length windows. Figure 5 depicts the resulting 54-point MDS three-dimensional map obtained with $\Delta_{NCD}$ for the period 1916–1969. We verify again the presence of the five periods $P_{1}$–$P_{5}$ and the inter-period tangle structures.

**Figure 6:**
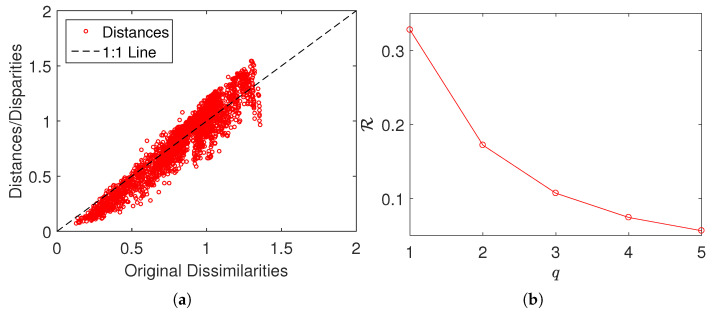
The MDS assessment charts obtained with $\Delta_{NCD}$: (**a**) Sheppard; (**b**) stress.

Figure 6a,b show the corresponding MDS assessment charts. Since the Shepard diagram reveals a small scatter around the 45 degree line, we conclude that there exists a good fit between the original and the reproduced distances. The stress plot shows that the three-dimensional map (n=3) is a good representation of the locus of points, since n=3 corresponds to the elbow of the curve. Therefore, three-dimensional maps represent a good compromise between accuracy and readability.

### 4.2. MDS Visualization of Complexity Based on the Euclidean Distance

**Figure 7:**
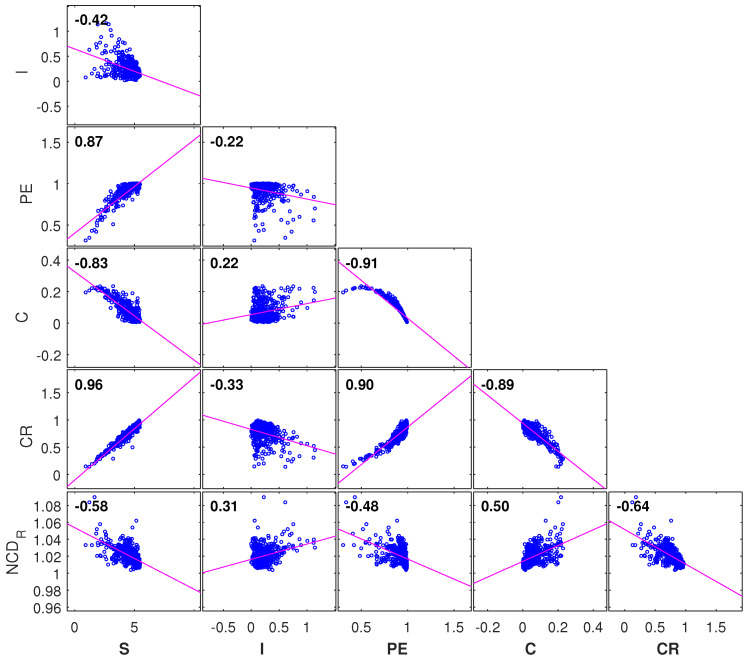
The correlation between the complexity indices $\left\{S,I,PE,C,CR,NCD_{R}\right\}$.

The six complexity indices $\left\{S,I,PE,C,CR,NCD_{R}\right\}$ reveal some correlation, as shown in Figure 7. Nonetheless, we conjecture that each index captures distinct details of the complex system and that a more complete assessment is accomplished when using all indices simultaneously. However, a six-dimensional visual representation is not possible and we decided to test the MDS technique for dimensionality-reduction and visualization.

In a first phase a 54×6 dimensional array, $T=[t_{ik}]$, is constructed, where $t_{ik}$, i=1,…,54, k=1,…,6, represents the *i*-th year and the *k*-th complexity index. The data in *T* is then normalized by the mean and standard deviation to avoid numerical saturation. Therefore, the columns of *T*, that is, $v_{k}$, are converted to:

(12) $${\hat{v}}_{k}=\frac{v_{k}-\mu \left(v_{k}\right)}{\sigma (v_{k})},$$

where μ(·) and σ(·) denote the arithmetic mean and the standard deviation, respectively. In a second phase, the lines of the normalized array, ${\hat{u}}_{i}$, are used for calculating the dissimilarity matrix $\Delta_{T}=\left[\delta \left({\hat{u}}_{i},{\hat{u}}_{j}\right)\right]$, i,j=1,…,54, where $\delta \left({\hat{u}}_{i},{\hat{u}}_{j}\right)={\left[\sum_{k}{\left({\hat{u}}_{ik}-{\hat{u}}_{jk}\right)}^{2}\right]}^{\frac{1}{2}}$ denotes the Euclidean distance between the complexity indices ${\hat{u}}_{i}$ and ${\hat{u}}_{j}$. Other distances can be adopted, but several numerical experiments that the Euclidean distance yields good results. Finally, in a third phase, the matrix $\Delta_{T}$ is processed by means of the MDS for constructing the loci of objects that represent the the evolution of complexity.

**Figure 8:**
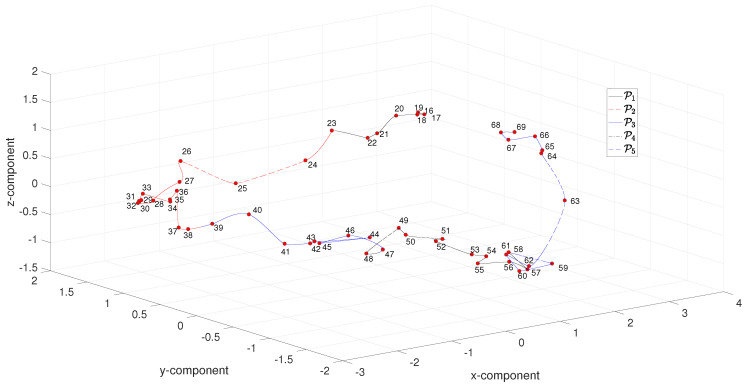
The MDS three-dimensional map obtained with $\Delta_{T}$ for the period 1916–1969.

Figure 8 depicts the MDS three-dimensional map obtained with $\Delta_{T}$ for the period 1916–1969. We verify roughly the same characteristics as before for the periods, but in this case we obtain a smoother trajectory, since the adoption of six indices works like a low-pass filter of noise present in the individual complexity indices.

The Sheppard and stress plots are omitted since they are similar to those shown in Figure 6a,b.

## 5. Conclusions

We adopted six complexity indices, dimensionality-reduction and visualization techniques for studying the evolution of Escher’s art. A total of 457 artworks, produced between years 1916 and 1969, were converted into digital format, discretized and represented in grayscale. The artworks were assessed by means of six distinct complexity indices and by the MDS technique. The results showed that the evolution of complexity is correlated with the periods of Escher’s artistic career. On a different level, we conclude that the proposed indices represent reliable and assertive tools for assessing artwork, and motivate their adoption in other artistic manifestations of the human spirit.
